# Iron Oxide Nanoparticles in Photothermal Therapy

**DOI:** 10.3390/molecules23071567

**Published:** 2018-06-28

**Authors:** Joan Estelrich, Maria Antònia Busquets

**Affiliations:** 1 Department of Pharmacy, Pharmaceutical Technology and Physical Chemistry, Faculty of Pharmacy and Food Sciences, University of Barcelona, Avda., Joan XXIII, 27–31, 08028 Barcelona, Catalonia, Spain; mabusquetsvinas@ub.edu; 2 Nstitut de Nanociència i Nanotecnologia, IN2UB, Facultat de Química, Diagonal 645, 08028 Barcelona, Catalonia, Spain

**Keywords:** photothermal therapy, photothermal agents, near infrared spectroscopy, magnetite nanoparticles, biological windows

## Abstract

Photothermal therapy is a kind of therapy based on increasing the temperature of tumoral cells above 42 °C. To this aim, cells must be illuminated with a laser, and the energy of the radiation is transformed in heat. Usually, the employed radiation belongs to the near-infrared radiation range. At this range, the absorption and scattering of the radiation by the body is minimal. Thus, tissues are almost transparent. To improve the efficacy and selectivity of the energy-to-heat transduction, a light-absorbing material, the photothermal agent, must be introduced into the tumor. At present, a vast array of compounds are available as photothermal agents. Among the substances used as photothermal agents, gold-based compounds are one of the most employed. However, the undefined toxicity of this metal hinders their clinical investigations in the long run. Magnetic nanoparticles are a good alternative for use as a photothermal agent in the treatment of tumors. Such nanoparticles, especially those formed by iron oxides, can be used in combination with other substances or used themselves as photothermal agents. The combination of magnetic nanoparticles with other photothermal agents adds more capabilities to the therapeutic system: the nanoparticles can be directed magnetically to the site of interest (the tumor) and their distribution in tumors and other organs can be imaged. When used alone, magnetic nanoparticles present, in theory, an important limitation: their molar absorption coefficient in the near infrared region is low. The controlled clustering of the nanoparticles can solve this drawback. In such conditions, the absorption of the indicated radiation is higher and the conversion of energy in heat is more efficient than in individual nanoparticles. On the other hand, it can be designed as a therapeutic system, in which the heat generated by magnetic nanoparticles after irradiation with infrared light can release a drug attached to the nanoparticles in a controlled manner. This form of targeted drug delivery seems to be a promising tool of chemo-phototherapy. Finally, the heating efficiency of iron oxide nanoparticles can be increased if the infrared radiation is combined with an alternating magnetic field.

## 1. Introduction

**Figure 1:**
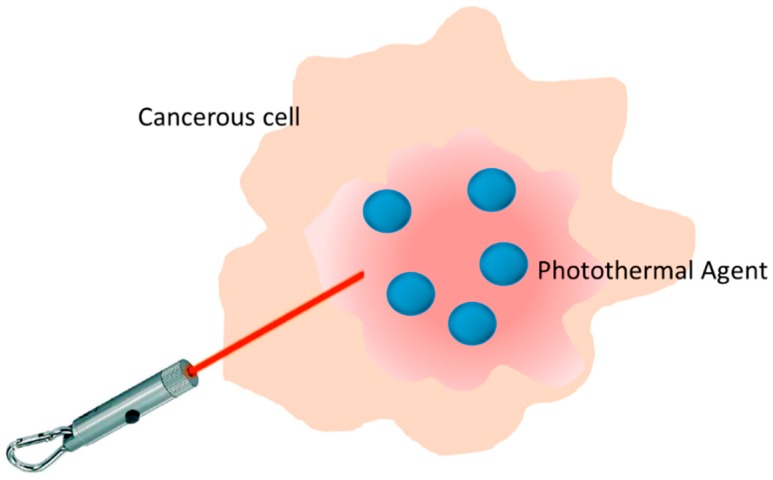
Basis of the photothermal therapy: the tumor containing the photothermal agents is irradiated with a laser. The radiation absorbed by the photothermal agents is converted to thermal energy causing cell death in the vicinity.

For a long time, heat has been known to be a potent way to destroy tissues, as burns testify every day. Thermal therapy is an approach of great interest in oncology, physiotherapy, urology, cardiology, and ophthalmology, as well as other areas of medicine. This kind of therapy includes two techniques, namely hyperthermia and thermal ablation. The difference between them is the threshold of temperature. In hyperthermia, the temperature rises up to 42 °C and it is maintained for a defined time; in thermal ablation, the temperature reaches more than 42 °C for a few minutes [1]. It has been demonstrated that the cancer cells can be killed after maintenance at 42 °C for 15–60 min; this duration can be shortened to 4–6 min for temperatures over 50 °C [2]. It is widely assumed that the efficacy of a thermal treatment is given by two main factors: the magnitude of the temperature increment and the duration of the treatment [3]. The role of thermal ablation in the management of cancer has gained increasing interest in recent years. Many different energy sources have been used for thermal ablation, including radiofrequency, high-intensity focused ultrasonography, microwave, alternating magnetic field, and laser [4]. Laser-induced photothermal ablation has been successfully employed for the ablation of tumors throughout the body, especially liver lesions. However, because of the heat-sink effect that dissipates heat and reduces the potency of the thermal effect, it is difficult to effectively treat lesions near large vascular structures using laser ablation alone [5]. Thus, the use of laser light-induced thermal ablation has been traditionally considered as a non-reliable technique, because human tissues show strong absorption coefficients in the visible range of the electromagnetic spectrum; this fact limiting photothermal treatments to superficial tumors [6,7]. In addition, the energy of a visible laser can be absorbed by both healthy and cancerous tissues, leading to a possibility of damage in non-cancerous tissues. To improve the efficacy and selectivity of laser-induced photothermal ablation, it is necessary to introduce light-absorbing materials, the photothermal agents (PA), into the tumor. This is the basis of the photothermal therapy (PTT). PA can convert absorbed light into heat, which results in the ablation of malignant tissue noninvasively by heating the tissue locally above 42 °C, while keeping the temperature of the surrounding tissue at a normal level (Figure 1).

**Figure 2:**
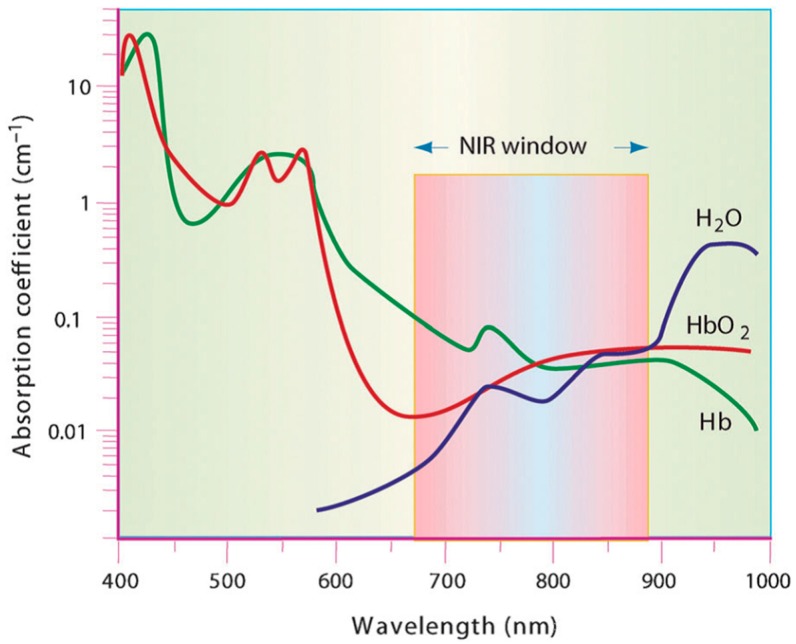
Absorption coefficients of hemoglobin (Hb), oxyhemoglobin (HbO_2_), and water. The absorption in the range of near-infrared (NIR) window is minimal. Reprinted with permission from Nat Biotechnol 19 (4), 316–317. Copyright 2001 [11].

PTT is an extension of photodynamic therapy (PDT), which involves the use of photosensitizing agents localized in tumor tissues in which a photosensitizer is excited with specific band light [8]. In PDT, upon activation, photosensitizers generate singlet oxygen that is acutely cytotoxic and causes irreversible free radical damage to tissues within a distance of approximately 20 nm [9]. Unlike PDT, PTT does not require oxygen to interact with the target cells or tissues. Moreover, the radiation used for exciting the photothermic materials is of longer wavelength, concretely the near-infrared (NIR) light range (from 650 nm to 1024 nm), and is therefore less harmful than that used in PDT to other cells and tissues [10]. Further reduction of non-desired light absorption by healthy tissues can be achieved using specific laser wavelengths lying in the so-called biological windows. Biological windows can be defined as the spectral ranges where tissues become partially transparent as a result of a simultaneous reduction in both absorption and scattering. Skin, tissues, and hemoglobin present minimal absorbance at the NIR range, especially for radiation with wavelengths ranging from 650 nm to 900 nm, with a peak of transmission at approximately 800 nm. This range of wavelengths of the electromagnetic spectrum is known as the first biological near-infrared window. In this, the radiation will penetrate more deeply into biological tissues than the visible wavelengths employed in photodynamic therapy. A second biological window extends from 1000 nm to 1350 nm, with both limits corresponding to water absorption bands. Figure 2 shows that hemoglobin and water, the major absorbers of visible and infrared light, respectively, have their lowest absorption coefficient in the NIR region around 650–900 nm [11].

### 1.1. Photothermal Agents

**Figure 3:**
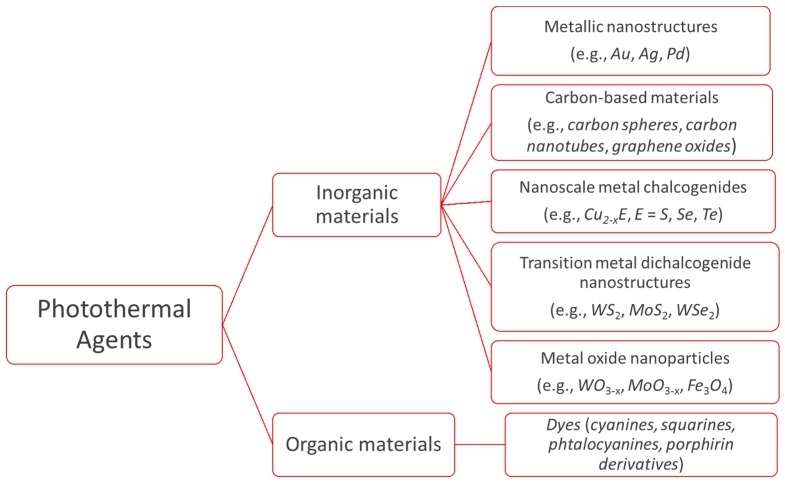
Classification of photothermal agents.

As indicated previously, PA are necessary to transform light into heat. A good PA must meet the following criteria: (i) minimal toxicity/maximal biocompatibility; (ii) diameter between 30 and 200 nm to promote long circulation and enhanced tumor accumulation; (iii) ability of absorb NIR radiation; and (iv) high absorption cross section to maximize light-to-heat conversion [10]. At present, a vast array of compounds of different composition, structure, shape, and surface coating are available as PA [12,13]. PA can be grouped into organic and inorganic materials (Figure 3) [14]. Inorganic materials comprise several types of substances, but the most used are metallic nanostructures and carbon-based materials. In the heterogeneous group of organic materials, organic dyes and polymer nanoparticles stand out. Jaque et al. [3] have provided a complete review of all the nanoparticles available for photothermal therapies up to 2014.

Metallic nanostructures, mainly referred to as plasmonics, hold a unique photophysical phenomenon, the local surface plasmon resonance (LSPR). When a particulate plasmonic material interacts with an electromagnetic radiation, the oscillating magnetic field of the radiation results in synchronized oscillation of the conduction-band electrons around the surface of the particles. This oscillation implies heat production. The amplitude of the oscillation reaches a maximum at a specific wavelength, called LSPR. One of the most tested examples is gold-based nanomaterials. Gold nanorods, nanoshells, nanostars, nanocages, and “cluster”-shape have been applied for treating tumor models in vivo, including photothermal ablation, targeted drug delivery, controlled drug release, and so forth [15]. Thanks to the diversity of nonmetallic materials, electronic transition materials are also being tested as PA. The capability of photothermal transduction is led from the electrons transition between molecular/atomic orbital energy levels, where the energy gap matches the light in NIR region.

Compared with inorganic PA, organic compounds, concretely NIR dyes, are very attractive candidates for PTT because their photophysical properties and availability for large-scale chemical synthesis [16]. Organic dyes also exhibit feasible conjugation with various kinds of specific molecules, such as chemical small molecules, amino acids, proteins, nucleotides, DNA primers, double-stranded DNA, and antibodies, which have been applied in molecular imaging [17].

The temperature rise in the tumor depends on the photothermal conversion efficiency (*η*) of the PA, the concentration of PA in the tumor, and the dosage of light delivered [10]. The photothermal conversion efficiency of any material used as PA is expressed as follows:

(1) $$\eta =\frac{hS\left(T_{\max}-T_{amb}\right)-Q_{s}}{I\left(1-10^{-A}\right)}$$

where *h* is the heat transfer coefficient, *S* is the surface area of the container, *T*_max_ is the maximum equilibrium temperature, *T_amb_* is the ambient temperature of the surroundings, *I* is the laser power, a is the absorbance of PA at the emission wavelength of the laser, and *Q_S_* is the heat associated with the absorbance of the solvent. When pure water is used as a solvent, *Q_S_* is 25.2 mW [18].

Once a PA to be used in PTT has been selected, it must be delivered to the tumor site either intravenously by targeted delivery or by direct injection, that is, intratumorally. When PA is administrated intravenously—the most realistic situation—the PA must be incorporated into the tumor by following two different strategies: active and passive targeting. For active targeting, the surface of the PA must be functionalized with a peptide or an antibody, which can be specifically recognized by proteins overexpressed in the tumor cells. This type of targeting is known as biological targeting. On the other hand, passive targeting is based on the enhanced permeability and retention effect (EPR) [19]. Because of abnormalities of tumor vasculature, nanoparticles in a certain size range (typically 20–300 nm) preferentially accumulate in tumor tissue. Thus, size significantly influences the pharmacokinetic behavior of PA in the body [20,21]. PA with large sizes (>40 nm at least in one dimension) are difficult to penetrate deeply in the tumor tissues and be cleared out by the body post-treatment, leading to decreased therapeutic outcomes and increased potential toxicity [22,23]. This problem can be addressed by reducing the particle size. It is demonstrated that sub-10 nm nanoparticles can penetrate into the deep region of the tumors, can be efficiently internalized by the tumor cells compared with the larger ones, and can also be rapidly cleared out of the body [24,25]. Photothermally induced cell death can take place via apoptosis or necrosis depending on the laser dosage, type, and irradiation time [12].

Up to the present, only preclinical experiments have been performed in the treatment of tumors with PA and laser irradiation. Such studies are in vitro experiments with cellular cultures and in vivo experiments with animal models, usually mice with a developed tumor. Despite the advantages of PTT against traditional chemotherapy and radiotherapy, PTT alone still faces the risk of tumor recurrence caused by invasiveness [26]. Consequently, PTT is combined with other treatments, for example, chemotherapy. This combination, named thermo-chemotherapy, achieves the release of chemotherapeutic drugs in a controlled manner through light and/or heat stimulus [27,28,29].

Consequently, thermo-chemotherapy greatly increases the concentration of drugs in the targeted cancerous area, and overcomes the safe dose limitation of drugs in the normal tissue. The synergistic effect of targeted heat delivery and controlled drug release has shown better efficacy in tumor treatment than PTT and chemotherapy alone. Furthermore, the capability of chemotherapeutic drugs to continuously destroy the remaining living cancerous cells after photothermal therapy makes thermo-chemotherapy a competitive candidate to simultaneously perform noninvasive tumor ablation and reduce the risk of tumor recurrence [30,31]. However, the method also presents drawbacks: the non-specific distribution of PA in normal tissues largely decreases their accumulation in a tumor, which limits the clinic applications of this strategy in cancer therapy. In consequence, the ability of selective targeting of PA is highly desirable for further enhancing of PTT efficacy and reducing side effects. Moreover, to supervise the distribution of the PA in tumors and other organs and monitor the therapeutic effect, imaging tools are also needed. As a result, the development of a theranostic system with targeting, therapeutic, and imaging capabilities has thus became important. In this way, nanoparticles that display the dual functions of magnetism and NIR absorption meet the above requirements. The magnetic feature offers the capacity of being used as magnetic resonance imaging (MRI) contrast agents, and the use of an external magnetic force allows for enriching the desired local tumor region with the nanoparticles, while they enable the transformation of NIR irradiation into heat for PTT.

### 1.2. Magnetic Nanoparticles: Iron Oxides

There are several types of magnetic particles with the ability to transform light into heat. For instance, magnetic ternary chalcogenide nanostructures, mainly those nanostructures focused on Cu–Fe–S, Cu–Co–S, and Cu–Fe–Se, have been employed as photothermal transducers [14]. However, among the magnetic particles with dual functions of magnetism and NIR absorption, iron oxide nanoparticles (IONs) stand out as nanoparticles suitable for PTT. IONs have great potential for use in biomedical applications because of their biocompatibility, biodegradability, facile synthesis, and ease with which they may be tuned and functionalized for specific applications [32]. Moreover, IONs have been approved for human use as MRI contrast agents. These properties, and the widespread acceptance of lack of toxicity, make IONs a good candidate to be used in cancer therapeutics via drug delivery, magnetic hyperthermia, photodynamic therapy, and photothermal ablation [33].

The purpose of this work is to provide a review of the use of magnetic nanoparticles for the treatment of tumors via photothermal therapies. We have focused the review on the IONs used to fight cancer tumors. After a brief description of IONs, their use in combination with other PA or alone is described. We highlighted especially those studies that show the application of IONs to both in vitro and in vivo thermal treatments and the best conditions to perform a specific PTT.

## 2. Iron Oxide Nanoparticles for Photothermal Therapy

Iron oxide can exist in different chemical compositions, such as magnetite (Fe_3_O_4_) or maghemite (γ-Fe_2_O_3_), or, most commonly, a non-stoichiometric combination of the two. Below certain sizes (25 nm for magnetite, 30 nm for maghemite), both oxides exhibit superparamagnetism behavior; that is, superparamagnetic nanoparticles become magnetic in the presence of an external magnet, but revert to a non-magnetic state when the external magnet is removed [34]. IONs with magnetic properties with a biocompatible coating have been exploited to enhance the contrast in magnetic resonance imaging (MRI) [35]. Moreover, the application of an external alternating magnetic field (AMF) to IONs leads to the production of energy, in the form of heat. Such effect can be considered in the use of IONs as mediators in magnetic hyperthermia [36]. At high AMF frequencies, the heat generated by the IONs is enough to produce temperatures above 42 °C. Sustained temperatures above 42.8 °C alter many of the structural and functional proteins within cells causing necrosis [37]. Another exploitation of the magnetic hyperthermia effect is for use as a drug-releasing trigger. If drugs, besides IONs, are encapsulated in a thermosensitive coating, the controlled release of drugs may be achieved when IONs heat up because of the application of an external magnetic field at relatively low frequency, for example, 50 kHz [38].

IONs can be used for PTT in two strategies: (a) IONs are combined with any PA forming hybrid nanoparticles that display the main functions of magnetism and NIR absorption (MRI, magnetic targeting, photoacoustic tomography, and PTT); and (b) IONs are used themselves as PA.

### 2.1. Hybrid Nanoparticles Formed with Iron Oxide Nanoparticles

IONs present a low molar absorption coefficient in the NIR region, and, in consequence, an apparent poor photothermal performance. For this reason, they are usually combined with other PA into a hybrid nanocomposite. Nanocomposites containing both magnetic particles and PA have attracted intensive attention for their biomedical applications as theranostic agents. As indicated previously, to guide the nanoparticles to the tumor side, surface modification of nanoparticles with a targeting ligand (e.g., peptide) is applied. As an alternative, which is different from biological targeting, other ways of targeting, that is, physical targeting, have been developed. One of them is the magnetic targeting: an external magnetic field can influence the movement of nanoparticles with magnetic properties. In this way, the combination of the functions of magnetization and NIR absorption into a single structure will make it possible for the resulting nanoparticles to be guided until the tumor, and they can work in MRI for visualizing the location of the cancer, or in thermal imaging for real-time monitoring of the treatment.

One of the more employed PA is gold-based nanomaterials. The strong cross-section absorption ability of gold nanostructures can generate adequate heat. Spherical gold-nanoparticles have not been very effective in vivo because these particles have peak absorptions belonging to visible light. For instance, a spherical gold-nanoparticle of 10 nm in diameter has a LSPR wavelength of 520 nm, compared with 580 nm for particles of 100 nm. However, the hierarchical assembly of gold-nanoparticles allows one to tune the LSPR wavelength to the NIR region. In this way, gold nanorods, nanoshells, nanostars, and nanocages have been used as PA for treating tumor models in vivo, because their LSPR is into the NIR region. Among the cited nanostructures, gold nanoshells, composed of a spherical silica core and a thin layer of gold (5–20 nm), have been found to have excellent photothermal therapeutic properties. Two such concentric spherical structures show red-shifted absorption due to the coupling between the inner and outer shell surface plasmons. Decreasing the ratio between the shell thickness and the core radius shifts the LSPR wavelength from visible to the NIR region. The LSPR wavelength shifts from 700 to 1000 nm when the shell thickness decreases from 20 to 5 nm. As an important handicap of gold structures, their metallic nature tends to scatter light, thus lowering the photothermal conversion efficiency [39].

Traditional designs of hybrid gold-IONs have been focused on building gold nanoshells around iron oxide cores. Coating the IONs’ surface with gold gives a hybrid nanostructure that combines MRI with PTT. There are two strategies for coating gold nanoshells onto IONs: the simple coating of gold on the IONs, and using a silica or polymer middle layer as the bridge of the magnetic core and the outer gold nanoshells. The simple coating of gold on the ION core limits the plasmon peak of the complex from 550 to 650 nm, rendering it unsuitable for PTT. Furthermore, these nanoparticles tend to form agglomerates losing potential applications. One of the first studies reported on the use, although indirect, of IONs in PDT was the paper of Larson et al. in 2007 [40]. They prepared nanoparticles formed with ION cores and gold shells for combined molecular specific MRI/optical imaging and photothermal therapy of cancer cells. The gold layer exhibited a surface plasmon resonance that provided optical contrast due to light scattering in the visible region. Moreover, the strong optical absorption of the plasmonic gold layer made these nanoparticles a promising agent for PTT. At the same time, the iron oxide cores gave a strong T_2_ contrast. The surface of the synthesized hybrid nanoparticles was functionalized with an antibody to specifically target the epidermal growth factor receptor (EGFR), a common biomarker for many epithelial cancers. Authors showed that receptor-mediated aggregation of anti-EGFR hybrid nanoparticles allowed for the selective destruction of highly proliferative cancer cells using a nanosecond pulsed laser at 700 nm wavelength. Similar studies were described by Ji et al. [41] and Hou et al. [42]. Core-shell type magnetic gold nanoparticles were also exploited to achieve the synergistic efficacy of radio-photothermal therapy in cervical cancer [43]. More complex structures are those formed by assembling gold nanorods, IONs, and gold nanoclusters in bovine serum albumin nanoparticles (BSA) [44].

A most direct application of the same hybrid nanoparticles was developed for their use in targeted photothermal destruction of colorectal cancer cells [45]. Such nanoparticles were functionalized with a single chain antibody for active targeting of the A33 antigen, which is overexpressed in colorectal cancer cells. The results showed that cells expressing the A33 antigen internalized the nanoparticles five times faster than cells not expressing the antigen. Furthermore, after six minutes of exposure to 808 nm laser radiation at a power density of 5.1 W·cm^−2^, 53% of A33-expressing cells died, while <5% of A33 non-expressing cells were killed. Flow cytometric analyses of the laser-irradiated A33 antigen-expressing cells show apoptosis-related cell death to be the primary mode of cell death at 5.1 W·cm^−2^, with increasing necrosis-related cell death at higher laser powers.

A kind of self-assembled nanostructures, derived from the above hybrid gold-IONs, are gold suprashells [46]. Gold suprashells have multiple surface plasmon resonances over a broad vis-NIR wavelength range. After excitation with two continuous wave lasers emitting light either 514 or 785 nm, such suprashells generated heat that could be used to kill cancer cells.

Most interesting is the use of a polymer middle layer between the magnetic core and the outer gold nanoshells. With this strategy, the relative dimensions between the magnetite core (e.g., diameter) and the gold shell (e.g., thickness) can be regulated, which allows for tuning of the plasmon resonance and the resulting optical absorption from 520 nm to 810 nm [4,41,47]. However, traditional silica gold nanoshells present two concerns that act as barriers for clinical applications: their size (140–150 nm) is not ideal for cellular endocytosis and tumor accumulation, and their difficult and time-consuming synthesis process. Another approach was employed by Zhang et al., which reported a sequential process involving sol-gel [48]. As an alternative to the silica middle layer, Hu et al. used a polyphosphazene derivative [49].

Similar, but more complex, hybrid nanoparticles have been used to treat B16–F10 melanoma tumors previously implanted on the right flank in mice [50]. For preparing these nanoparticles, IONs were conjugated onto silver cores using 3-mercaptopropyltrimethoxysilane. Then, a second layer of silver was formed over the IONs and reduced with hydroxylamine. When gold salt was added to the silver complex, a thin gold layer was formed while etching away the silver. This novel theranostic nanoparticle, formed by a magnetic hollow gold nanoshell, combines the optical and photothermal properties of hollow gold nanoshell nanoparticles with the magnetic properties of IONs, while maintaining dimensions in the 40–80 nm range. The nanoparticles demonstrate to be effective PTT agents. Tsai et al. have prepared nanoparticles made with a double layer of gold/silver alloy on the surface of truncated octahedral IONs [51]. If the distance between the layer is well controlled, the nanoparticles can exhibit a broad and strong NIR absorption. Other gold-shelled nanoparticles were prepared by other groups [52,53,54,55,56,57].

One concern of the plasmonic–magnetic hybrid nanoparticles is that the relaxivity of the magnetic component is reduced by the plasmonic component in conventional core-shell structure nanoparticles. Liu et al. prepared yolk-shell structured magnetic–plasmonic nanoparticles comprising a magnetite core within a hollow cavity encircled by a porous gold outer shell [58]. The introduction of the hollow cavity between the magnetic and plasmonic portions prevented the decline in relaxivity. Moreover, in addition to conferring high NIR absorption to the plasmonic component, the hollow cavity and the pores in the outer shell can provide storage space and release channels for anticancer drugs. Shen et al. also prepared nanoparticles with thermosensitive yolk-shell structures [59].

Most of the gold/IONs hybrid nanoparticles involve gold nanospheres or gold nanoshells. Few works focused on the composites of gold nanorods and IONs [60,61,62,63], because it is quite difficult for gold nanorods to grow directly on the magnetite surfaces. Recently, Hu et al. have prepared ternary assemblies of negatively charged magnetite cores, polycation modified gold nanorods, and polycations [64]. The prepared multifunctional nanocomposites can also complex plasmid DNA. The multifunctional applications of these nanocomposites in trimodal imaging (MRI, photoacoustic imaging, and computed tomography) and combined PTT/gene therapy were corroborated using a xenografted rat glioma nude mouse model. Redolfi et al. have also used nanorods [65]. Their strategy is based on the electrostatical assembly of negatively charged gold nanorods on IONs coated with positively charged silica. Finally, the biological stability was increased by coating the assembly with BSA. Other hybrid nanoparticles formed by gold nanopopcorns [66] or nanostars [67,68] containing a self-assembled iron oxide cluster core emerged. Hybrid nanostructures formed by hollow gold nanospheres and IONs were also described [69]. Recently, complex structures have been gradually formed by several cycles resulting in ferroferric oxide@dye/silica@gold nanoshells [70].

A mesoporous magnetic gold nanocluster with a high drug loading capacity has also been used for PTT [71]. In this study, doxorubicin (DOX) was the encapsulated drug. After NIR laser irradiation, the temperature of the nanoparticle dispersion reached 52 °C in 5 min. By in vitro cytotoxicity testing, the combination of PTT and chemotherapy caused more damage that chemotherapy or PTT did alone. Moreover, such nanoparticles could be targeted to the tumor of mice when submitted to an external magnetic field (magnetic targeting).

IONs can be combined with other inorganic nanoparticles such as molybdenum sulfide [72,73], manganese [74], copper/selenium [75,76], graphene oxides [77,78], manganese and graphene oxide [79], Prussian blue [80,81], and copper sulfide [82].

Wang et al. described a more complex structure composed of a core of magnetite, a porous carbon shell, where fluorescent carbon dots were embedded and coated with gold [83]. The porous carbon shell endowed the nanoparticles with excellent stability in the aqueous phase and a high loading capacity for the anti-cancer drug DOX. The combined photothermal effects of the gold nanocrystals and the carbon dots in the carbon shell can not only regulate the release rate of the loaded drug, but also efficiently kill tumor cells under NIR irradiation.

A system formed by IONs as a core, mesoporous silica as a shell, fucoidan, a marine biopolymer coated onto the outer surface, and DOX showed to be an excellent system for pH-stimuli responsive drug releasing [84].

**Figure 4:**
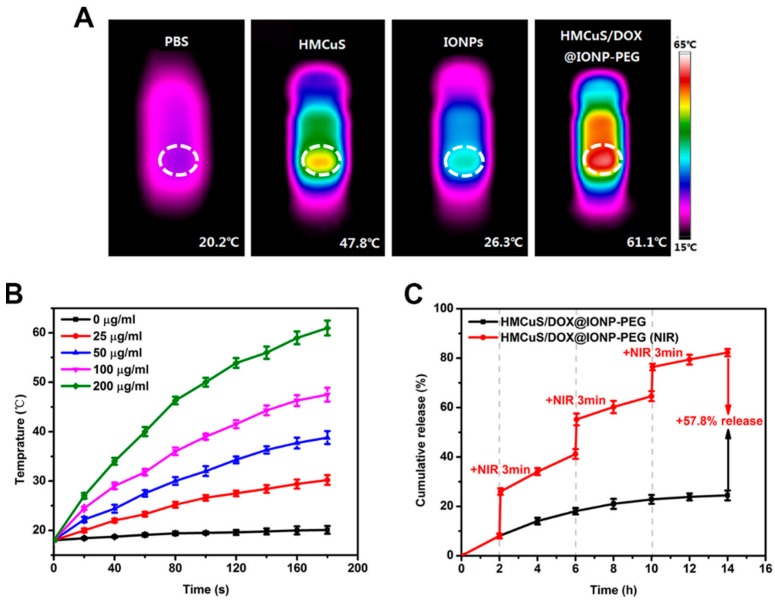
Photothermal heating effect and in vitro release profiles. (**A**) IR thermal images of tubes with phosphate buffer (PBS), hollow mesoporous CuS nanoparticles (HMCuS), iron oxide nanoparticles (IONs) and HMCuS/DOX@ION-polyethylene glycol (PEG) after irradiation by a 808 nm laser for 3 min; (**B**) the photothermal heating curves of HMCuS/DOX@ION-PEG with different concentrations after irradiation by an 808 nm laser; (**C**) release curves of doxorubicin (DOX) from the hybrid system with or without NIR irradiation. Reprinted with permission from Acta Biomater, 49, 402–413. Copyright 2016 [85].

Feng et al. developed a system by capping DOX-loaded hollow mesoporous CuS nanoparticles (HMCuS) with IONs and coated with polyethylene glycol (PEG) (HMCuS/DOX@ION-PEG) [85]. Such a hybrid system can be used as either a photothermal or photodynamic agent, because the photothermal conversion efficiency was 42.12%, and the OH^●^ generation was demonstrated under NIR irradiation. The photothermal effect is depicted in Figure 4.

In Figure 4A, we can evidence the temperature achieved after 3 min of irradiation by a laser of 808 nm in phosphate buffer (PBS) (20.2 °C), HMCuS (100 µg·mL^−1^) (47.8 °C), IONs (20 µg·mL^−1^) (26.3 °C), and HMCuS/DOX@ION-PEG (200 µg·mL^−1^, containing 100 µg·mL^−1^ HMCuS and 20 µg·mL^−1^ IONs) (61.1 °C). Figure 4B revealed that the photothermal effect occurred in both concentration- and time-dependent manners. The capping of IONs onto hollow mesoporous nanoparticles avoided the premature release of the drug and, consequently, its premature leakage in circulation. Moreover, after NIR irradiation, some ION-caps could be remotely removed from the surface of HMCuS/DOX@ION-PEG and, in this way, controlled on-demand drug release with spatial/temporal resolution was produced. This can be observed in Figure 4C. The no-laser treated group displayed a sustained-release property along with the time progress and only a small amount of DOX (24.4%) released within 14 h. By contrast, a burst release of DOX occurred with NIR irradiation, indicating an NIR-responsive controlled drug release profile in an impulsive manner.

The synergic effects of PTT, PDT, and chemotherapy were explored in breast cancer cells and in mice. In the study in vivo, the nanoparticles under IR irradiation reduced the tumor growth at the initial level. Moreover, after applying the magnetic field, the accumulation of nanoparticles was higher and the tumors were almost completely suppressed recurrence.

An alternative strategy to the use of conventional inorganic PA was to incorporate an organic PA. Organic-based PA have greater potential for in vivo applications because of their better biocompatibility and biodegradability [86]. Usually, IONs can be combined with NIR dyes. Such dyes have strong absorption in the optical biological windows, which may facilitate the PTT efficacy and deep penetration of the light into the tissue. Ma et al., for instance, produced a nanocomposite that induced intense temperature elevation and tumor ablation upon laser irradiation by loading indocyanine green (ICG) into 1,2-distearoyl-sn-glycero-3-phosphoethanolamine-*N*-[methoxy (polyethylene glycol)] (DSPE–PEG) coated IONs (ION@DSPE-PEG/ICG) [87]. ICG is an FDA-approved substance for a number of clinical imaging applications, and it shows an appreciable light-to-heat conversion efficiency. Encapsulation of ICG in ION@DSPE-PEG resulted in higher photostability than free ICG because of the protection from degradation.

Song et al. reported a new nanocomposite based on another cyanine, the heptamethine indocyanine (that absorbs at 825 nm), which, in the presence of a cationic polymer, forms J-aggregates with red-shifted and significantly enhanced absorbance at ~915 nm [88]. After complexing with IONPs and further surface PEGylation, the resulting nanocomposite could be utilized for in vivo MRI-guided PTT triggered at 915 nm, which interestingly appeared to be optimal in PTT applications, because of its improved tissue penetration when compared with a radiation of 808 nm, and much lower water heating in comparison with a radiation of 980 nm.

**Figure 5:**
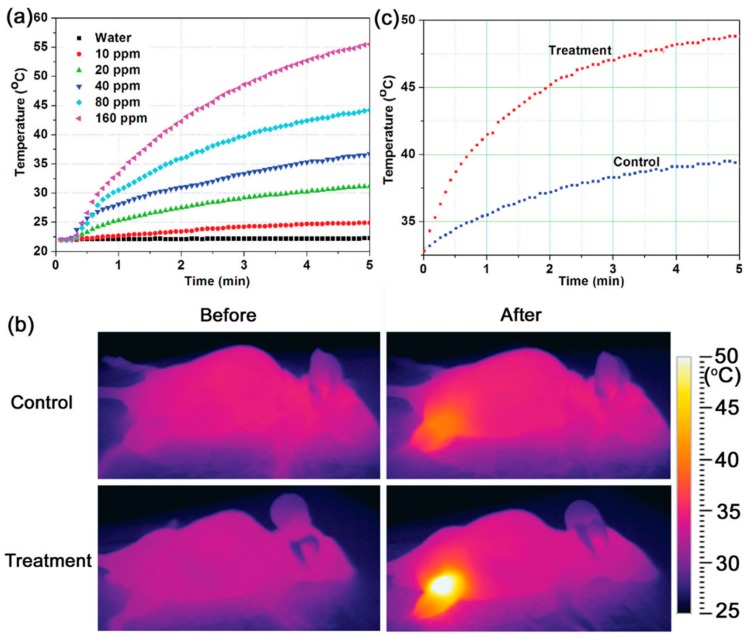
(**a**) Temperature profiles of pure water and aqueous dispersions of ION@polypyrrole (PPY) with different particle concentrations (10, 20, 40, 80, and 160 ppm) as a function of irradiation time; (**b**) infrared thermal images of the tumor-bearing mice injected with 100 µL of phosphate buffer (control) and 80 ppm ION@PPY (treatment) before and after irradiation for 5 min; (**c**) the temperature profiles of the tumor (irradiated) regions in the control and the treatment as a function of the irradiation time. An 808 nm laser with a power density of 0.25 W·cm^−2^ was used for the irradiation. Reprinted with permission from Small 10 (6), 1063–1068. Copyright 2014 [90].

Other hybrid systems are formed by polypyrrole (PPY). This compound has demonstrated to be a biocompatible PA with high photothermal conversion efficiency and good photostability [89]. It is interesting to note that Fe^3+^ ions are used as the oxidation agents for the preparation of PPY nanoparticles. Consequently, a large number of ferric (Fe^3+^) and ferrous ions (Fe^2+^) remain in the obtained PPY nanoparticles. Tian et al. utilized these residual Fe ions as precursors to produce magnetite crystals in situ on the surface of pre-synthesized PPY nanoparticles [90]. The resulting ION@PPY composite nanoparticles integrated MRI imaging, infrared thermal imaging, and photothermal conversion functions, and they exhibited excellent therapeutic effectiveness against cancer, as demonstrated in both in vitro and in vivo studies. They observed that after continuous irradiation of an 808 nm laser with a power density of 0.25 W·cm^−2^ for 5 min, the temperature of an aqueous dispersion of the ION@PPY increased by ≈2.9–33.5 °C, depending on the concentration of the nanoparticles. The same treatment resulted in a negligible temperature increase (<0.3 °C) when pure water was used as the control (Figure 4A). This property allowed ION@PPY to act as in vivo thermal imaging contrast agents. The spatial temperature distribution in the tumor-bearing mice was detected in real-time using an infrared thermal imaging system after intratumoral injection of PPY@ION. It was found that upon the laser irradiation, the tumor region of the mice in the treatment group heated up very quickly, with the temperature reaching 48.8 from 32.8 °C in 5 min (Figure 5c), and consequently, a strong image contrast was observed (Figure 5b). In comparison, the control group reached a temperature of 39.2 °C after the same period of irradiation (Figure 5c) and produced a much weaker image contrast (Figure 5b).

These results suggested an added benefit of ION@PPY, that is, they allow for real-time monitoring of the temperature dynamics of a photothermal therapy process by infrared thermal imaging. More importantly, these nanoparticles could efficiently kill cancer cells in both in vitro and in vivo experiments in a short time (<5 min).

A similar type of magnetic targeting used for combined PTT and chemotherapy was reported: DOX delivery in the presence of magnetically promoted cellular uptake with NIR-absorbing PPY-coated IONs [91]. Zhang et al. also fabricated IONs enveloped with PPY [92]. Recently, Han et al. have constructed a nanostructure composed of PPY-coated IONs and a gold nanoshell, which is capable of enhancing the photothermal therapeutic effect and the contrast for both MR and X-ray computed tomography imaging [93].

Chen et al. used both inorganic (e.g., gold sulfide nanoparticles) and organic materials (e.g., IR820 dye) to decorate polysiloxane-containing polymer-coated IONs [94]. Chang et al. anchored IR806 dye on the surface of IONs and introduced citraconic anhydride as a smart charge-conversion agent to improve the targeted accumulation in the tumor [95].

Another organic substance that can decorate IONs is polydopamine (PDA), a melanin-like mimic of mussel adhesive proteins [96,97,98,99]. PDA fulfills all the requirements regarding a versatile functional coating for multimodal theranostic materials [100]. For instance, Wu et al. used nanoparticles composed of an iron oxide nanocluster core coated of PDA, further conjugated with PEG and adsorbing ICG on the surface. The adsorbed dye in the nanobead displayed a higher photostability and photothermal conversion ability than free dye, as well as an additional photothermal effect, rather than magnetite nanocluster and PDA [101]. Peng et al. described a similar work [102]. Sun et al. synthesized a magnetic nanosystem comprising a core of ION, a PEGylated shell of PDA, and loaded with ICG and DOX [103]. As a tumor-specific environment cue, the acidic condition was able to trigger a burst mode of drug release from the nanocarrier. Liu et al. coated IONs with PDA and then conjugated the nanoparticles with an affibody (a non-antibody protein) of high affinity to tumor [104]. As indicated above, IONs can be coated with PDA and then loaded with DOX. The resultant product showed that the combination of chemotherapy and PTT had an evident synergistic effect on the ablation of tumor cells [105]. Moreover, to increase the selectivity of the targeting, an EGFR antibody can be located on the surface of these nanoparticles [106]. IONs coated with PDA can be also modified with 6-thio-*β*-cyclodextrin and loaded with DOX [107].

In a similar way, Sun et al. designed a magnetic nanosystem formed by a core of IONs, and a PEGylated shell of polydopamine loading ICG and DOX. The system, which can be guided magnetically, is suitable for combining PTT, photodynamic therapy (PDT), and chemotherapy. Experimental studies based on HeLa cells showed remarkable effects on cell viability, apoptosis, and uptake efficiency [103]. Using the same cyanine, a nanoplatform was obtained by in situ growth of IONs on carbon nanoparticles, and then loaded with the dye [108].

IONs have also been used in mitochondrial-targeting therapy [109]. With this aim, Wang et al. have prepared IONs containing PDA [85]. In this study, a multistage targeting strategy using DOX-loaded magnetic composite nanoparticles has been developed for enhanced cancer treatment. The nanoparticles with a core-shell-S-S-shell architecture are composed of a core of IONs clusters, an inner shell of PDA functionalized with triphenylphosphonium (TPP), and an outer shell of PEG linked to the PDA. After the nanoparticles have entered the tumor cells, the stage of mitochondrial targeting is realized as the PEG shell is detached from the particles by redox responsiveness to exposure to the TPP. After irradiation with a near-IR laser at the tumor site, a photothermal effect is produced, leading to an important decrease in mitochondrial membrane potential. Then, the loaded DOX can easily enter the mitochondria and damage the mitochondrial DNA, resulting in cell apoptosis.

The possibilities of combining IONs with chemical compounds are numerous. In this way, Yang et al. combined IONs with metalla-aromatic agents (aromatic rings in which the elements such as carbon, nitrogen, oxygen, or sulfur are replaced with transition metal atoms) and loaded them inside a micellar carrier [110]. The resulting nanocomposite presented an important photothermal transduction efficiency (26.6%) and was, furthermore, a photodynamic sensitizer. The combined photothermal and photodynamic therapy achieved a synergistic anti-tumor effect.

### 2.2. Iron Oxide Nanoparticles as Photothermal Agents Themselves

**Figure 6:**
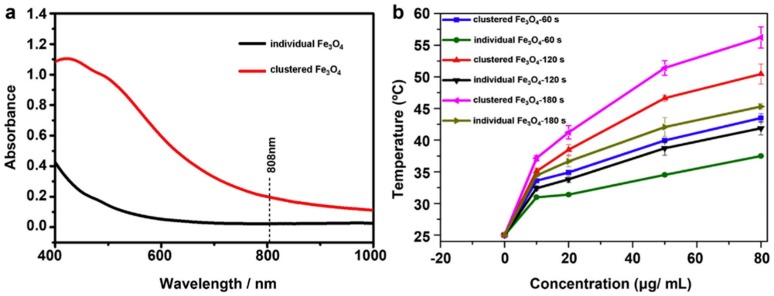
(**a**) Ultraviolet visible (UV-vis) spectra of aqueous dispersion of individual and clustered IONs; (**b**) changes of temperature underwent by the suspensions of individual and clustered IONs (0–80 µg·mL^−1^) after irradiating them for 60–180 s. Reprinted with permission from Biomaterials 39, 67–74. Copyright 2015 [112].

IONs can be used as PA without decoration with gold or other photothermal agents. IONs have demonstrated to be effective PA in various applications. For instance, they can drive high barrier reactions, such as the clean decomposition of propylene carbonate [111]. Concerning biomedical applications, the intrinsic ability to generate heat within a short time under NIR laser irradiation makes IONs a novel promising hyperthermia agent. However, it is stated that when IONs are used as agents for NIR light-induced PTT by themselves, high irradiation density of light (over 1 W·cm^−2^) is usually required for effective ablation of the tumor [112]. This irradiation by far exceeds the safe limit for cutaneous tissues (0.33 W·cm^−2^ for an 808 nm laser) [113]. This drawback seems to affect IONs when they are in individual form, as one study has reported the importance of the aggregation state of IONs in relation to the photothermal effect [112]. The authors compared the photothermal effect of clustered IONs with individual IONs and observed a significant ~3.6-fold increase in the NIR absorption at 808 nm in the aggregated nanoparticles (Figure 6a). After an irradiation at 808 nm and power density 5 W·cm^−2^ for several times, the increase of temperature was determined. Clustered IONs were more efficient than the individual IONs in inducing a temperature increase with the same concentration (Figure 6b).

The cytotoxic effects produced by the IONs (at the concentration of 50 µg·mL^−1^) and further NIR irradiation (power density of 5 W·cm^−2^) were determined for different illumination times of 60, 120, and 180 s. Approximately 8.9%, 33.5%, and 72.8%, respectively, of the A549 cells (human lung adenocarcinoma epithelial cells) were killed by clustered IONs, and only 0.8%, 3.5%, and 14.5%, respectively, of the cells were killed by individual IONs. The photothermal ablation of tumors in vivo was performed in A549 tumor-bearing mice. After subcutaneous injection of tumor cells into the animals’ flank region, tumors developed. When the size of tumors achieved ca. 5.0 mm in the longest dimension, the animals were randomized. Three groups intratumorally received the nanoparticles. Twenty-four hours later, mice were irradiated with a 808 nm laser (5 W·cm^−2^ for different times, 120 s or 180 s, according the experimental group). A statistically significant reduction of the tumor volume was observed in those mice treated with clustered IONs.

In two studies, Guo et al. synthesized monodisperse IONs from 10 to 310 nm and studied the behavior and magnet-assisted antitumor efficacy [114,115]. There were no significant differences among nanoparticles in the 60–310 nm size range in photothermal conversion efficiency (808 nm, 1.5 W·cm^−2^, 10 min). In the in vivo anticancer efficacy studies, mice-bearing tumors were injected with DOX-loaded IONs, and then irradiated with NIR (808 nm, 1.5 W·cm^−2^ for 3 min, 3 irradiations). At any case, the tumor temperature rose quickly. However, IONs of 310 nm generated the highest increase. This is due to the fact that the nanoparticles exhibited the highest accumulation at the tumor site.

Another strategy is to prepare positively charged IONs [116]. These nanoparticles bind electrostatically cancer cells that bear negative charges on their surface. The further irradiation with an 808 nm laser induces photothermal effects.

In other studies, IONs are complexed with other substances. For instance, Liao et al. synthesized iron oxide nanostructures by the introduction of benzene-1,3,5-tricarboxylic acid and sodium citrate as co-coordinating agents [117]. The presence of the complex on the surface of IONs led to a ligand-induced surface effect, which promoted the large transition in the NIR wavelength from d–d transitions of iron ions. The resulting IONs were further coated with mesoporous silica to achieve sufficient dispersion. When exposing such nanoparticles to irradiation of 808 nm with a power density of 2 W·cm^−2^, the temperature increased to 44 °C in 4 min. In the in vitro test, a significant phototoxicity of human oral carcinoma KB cells treated with the nanoparticles was observed after an 808 nm laser exposure for 15 min at a power density of 1.4–2 W·cm^−2^. In the in vivo study, mice bearing the indicated tumor were injected intratumorally with the nanoparticles (0.5 mg·kg^−1^). After NIR irradiation (2 W·cm^−2^ for 10 min), the temperature of the tumor increased to 47 °C from 34 °C. Song et al. utilized tannic acid and IONs to construct smart magnetic assemblies based on polyphenols [118]. Such assemblies were disassembled by ATP, which showed a stronger affinity by the acidic environment. The assemblies can be used in PTT. IONs can also be combined with graphene [119] and modified with hyaluronic acid [120].

Human serum albumin (HSA), the most abundant plasma protein, is a natural transport vehicle with multiple ligand sites. Hai et al. covalently conjugated HSA onto IONs [121]. The therapeutic potential of this hybrid for PTT was demonstrated using a laser of 808 nm (1 W·cm^−2^). At the lowest iron concentration used (2.5 mM), the temperature increment after 300 s of exposure was 9.2 °C, whereas at the highest concentration (6.5 mM), it was 14.1 °C. Then, the photothermic effect was tested on two cell lines (HeLa and murine fibroblasts). After 500 s of treatment (808 nm, 1 W·cm^−2^, iron concentration = 2.5 mM), it was observed that apoptosis was triggered for cancer cells, but not for fibroblasts.

Contrary to the affirmation that a certain extent of aggregation of IONs is necessary to achieve a photothermal effect at safe irradiation doses, Chen et al. found that highly crystallized IONs with a delicate polymer coating could be used for efficient photothermal ablation [122]. In another study, Chu et al. investigated the photothermal effect of IONs of several shapes (spherical, hexagonal, and wire-like) coated with carboxyl-terminated PEG-phospholipid [123]. When the IONs (0.5 mg·mL^−1^) were taken up by Eca-109 cells (human esophagus carcinoma cells), the viability and cell structure was not affected. Upon irradiation with a laser of 808 nm however, the cell viability was suppressed (<60% in the three types of IONs). For an in vivo photothermal therapy, nude mice subcutaneously transplanted with human esophageal tumors on their right flanks were used. Tumors were directly injected with 70 µL of IONs (magnetite: 8 mg·mL^−1^), followed by irradiation with the 808 nm laser for 20 min every 24 h for 24 days. After this time, the tumor growth was significantly suppressed.

The application of IONs in vivo has been strongly hampered by high macrophage uptake, short blood retention time, and unfavorable biodistribution. Thereby, the stabilization of IONs in a physiological environment requires the coating of the particles with a polymer. Carboxymethyl chitosan is an example of polymer used to stabilize IONs [124]. These nanoparticles exhibited a comparable photothermal effect to that obtained with hollow gold nanospheres. The in vivo MR images of mice revealed that by attaching a magnet to the tumor, the particles accumulated in the tumor after intravenous injection and showed a low distribution in the liver. After being exposed to an 808 nm laser for 5 min at 1.5 W·cm^−2^, the tumors were completely destroyed. Another coating polymer is the poly(acrylic acid) [125]. The current gold standard method to reduce the uptake by the mononuclear phagocyte system is a surface modification of nanoparticles with a layer of PEG. In this way, IONs coated with PEG have been used for in vitro cancer treatment of C6 cells. After exposition to an 808 nm laser with an intensity of 2 W·cm^−2^ for 5 min, nearly all of the C6 cells, previously incubated with IONs for half an hour, were killed [126]. In another study, the cyclic peptide arginine-glycine-aspartic acid-d-tyrosine-lysine (cRGDyK) was conjugated to PEG-IONs to achieve a tumor-targeting multifunctional nanotheranostic agent [127]. This peptide is a specific ligand for the receptor αVβ3 integrin, overexpressed in tumor cells. More recently, clusters of IONs were camouflaged into red blood cell membranes [128]. However, some reports have demonstrated that PEG triggers an “accelerated blood clearance” (ACB) phenomenon in experimental animals: a second dose of PEGylated nanoparticles given several days after the first injection was rapidly cleared out from blood circulation [129]. Moreover, recent studies have indicated that the presence of PEG in liposomal surfaces reduces their interaction with cells [130]. To alleviate the ABC effect, red blood cell (RBC) membranes can be used as biomimetic coating for nanoparticles [131]. There are several methods to prepare RBC membrane-coated nanoparticles, although electroporation is one of the most facile and effective [132]. This strategy relies on applied electric fields to break down the dielectric layer over cell membranes and create multiple transient pores for biomolecules and nanoparticles to enter [133]. Using this strategy, Rao et al. synthesized RBC-membrane-coated IONs that were used in PTT [134], as well as platelet-membrane-coated IONs [135]. Other IONs have been coated with meso-2,3-dimercaptosuccinic acid (DMSA) and loaded with DOX [109].

Any magnetic particle can be directed by an external magnetic field until a desired zone. This promising targeting tool allows enriching magnetic nanoparticles in the desired local tumor region during their circulation in the blood. Based on this physical property, Li et al. used a magnetic field to enhance the effect of IONs in PTT [136]. IONs were functionalized with PEG using a grafted polymer, dopamine–polyacrylic acid-PEG. Such IONs were loaded with chlorin e6, a photosensizer used in PDT. The complex exhibited a red shift in the absorbance peak of chlorin e6 (from ~650 nm to 700 nm). In the presence of an external magnetic field, 4T1 murine breast cancer cells incubated with the nanoparticles followed by 704 nm laser irradiation with a power density of 5 mW·cm^−2^ exhibited 90% photothermal cell destruction. In addition, of the cells incubated with the same concentration of nanoparticles followed by 660 nm irradiation with a 5 mW·cm^−2^, only ~30% of the cells were destroyed. For the in vivo experiment, the mice bearing 4T1 cell tumors were intravenously injected with the nanoparticles under the external magnetic field and irradiated with a 704 nm laser (at 5 mW·cm^−2^). The volume of the tumor remained controlled for 16 days, whereas in the control group the tumor size increased.

Zhou et al. have prepared PEGylated iron/iron oxide core/shell nanoparticles (Fe@ION) [137]. Such nanoparticles possess triple functional properties in one entity—targeting, PTT, and imaging. Compared with gold nanorods, they exhibit similar photothermal conversion efficiency (~20%), and much higher photothermal stability. In the in vitro study, HeLa cells were incubated with Fe@ION for 1 h in the absence or presence of a magnet (0.5 T) beside the cells. Then, cells were irradiated by an 808 nm laser (0.31 W·cm^−2^) for 10 min. Nearly all of the cells that were incubated with the nanoparticles and laser irradiated underwent the ablation, while for the control group, only a few dead cells were observed. For in vivo photothermal imaging, nanoparticles were injected intravenously into HeLa tumor-bearing mice. After the injection, mice of the magnetic targeting group were set beside the indicated magnet for 12 h, and then were exposed to an 808 nm laser for 10 min [5]. The results showed that the external magnetic field promoted the accumulation of nanoparticles in the tumor region, indicating the good magnetic targeting ability. In the tumor, the increased temperature was approximately two times that recorded without magnetic targeting. Notably, the intrinsic high photothermal conversion efficiency and selective magnetic targeting effect of the nanoparticles in the tumor play synergistically in highly efficient ablation of cancer cells in vivo and in vitro.

**Figure 7:**
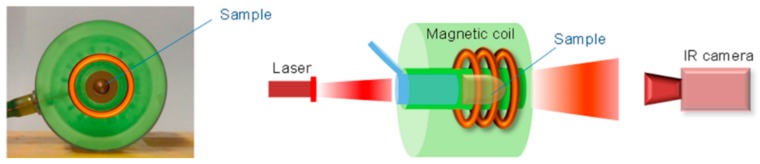
Scheme of the experimental device for combined hyperthermia experiments consisting of a magnetic coil in which the sample is placed, so that it can be illuminated by the NIR laser. The temperature increase was recorded with an infrared thermal image camera located at the end of the coil cavity. Reprinted with permission from ACS Nano 10, 2, 2436–2446. Copyright 2016. American Chemical Society [138].

The heating efficiency of IONs can be amplified by magnetic hyperthermia and photothermal bimodal treatment. Espinosa et al. have used nanocubes of iron oxides, which were exposed to both an alternating magnetic field and NIR laser irradiation (Figure 7) [138].

**Figure 8:**
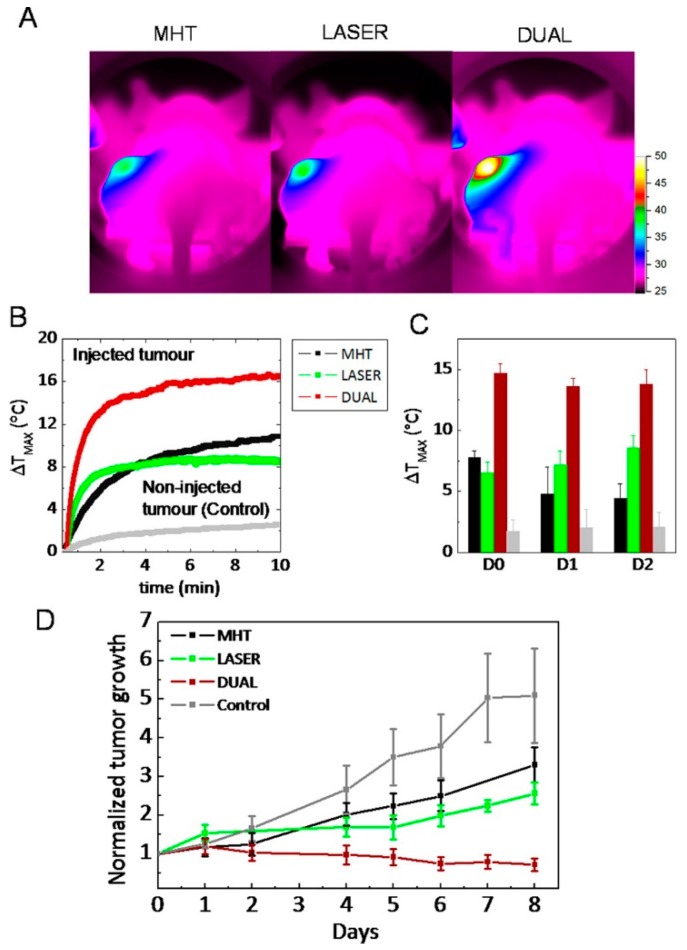
In vivo heat therapy. (**A**) Thermal images obtained with the IR camera in mice, after intratumoral injection of nanocubes (50 µL at [Fe] = 250 mM), in the left-hand tumor, and after 10 min application of magnetic hyperthermia (MHT, 110 kHz, 12 mT), NIR-laser irradiation (LASER, 808 nm at 0.3 W·cm^−2^), or DUAL (both effects); (**B**) corresponding thermal elevation curves for all treatments and for the noninjected tumor in the DUAL condition; (**C**) average final temperature increase obtained after 10 min (MHT, LASER, and DUAL) on day 0 (1 h after injection) and 1 and 2 days after injection and for non-injected tumors; (**D**) average tumor growth groups of six tumors each in non-injected mice submitted to no treatment (Control) and in nanocube-injected mice exposed to MHT, LASER, and DUAL during the 8 days following the 3 days of treatment. Reprinted with permission from ACS Nano 10, 2, 2436–2446. Copyright 2016. American Chemical Society [138].

This treatment, which was carried out at compatible clinical doses with a low iron concentration (250 mM) and acceptable low irradiation (0.3 W·cm^−2^), amplified the heating effect 2- to 5-fold in comparison with magnetic stimulation alone. The dual heating effect was tested in environments of increasing biological complexity from aqueous suspensions to tumor cells in vitro and solid tumors in vivo. In all these situations, the two heating effects were cumulative, if not synergistic, yielding a high heating power (5000 W·g^−1^). In cancer cells, the laser restored the optimal efficiency of magnetic hyperthermia, otherwise inhibited by cellular confinement. Consequently, the dual action yielded complete apoptosis-mediated cell death. In solid tumors in vivo, single-mode treatments (magnetic or laser hyperthermia) reduced tumor growth, while this dual treatment resulted in complete tumor regression, mediated by heat-induced tumoral cell apoptosis and massive denaturation of the collagen fibers, and a long-lasting thermal efficiency over repeated treatments (Figure 8).

**Figure 9:**
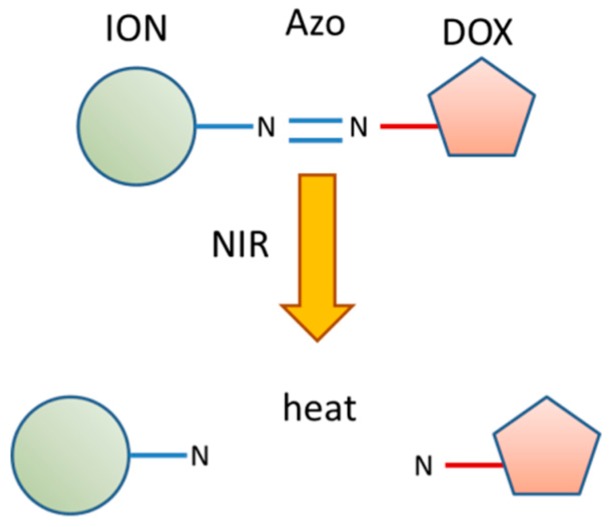
Schema of the breakage of IONs–4,4-azobis (4-cyanovaleric acid) (azo)–DOX after irradiation with NIR laser.

Functionalized IONs can combine chemo-photothermal therapy and targeted drug delivery. In order to achieve a better antitumor effect, it is important that the drug is released in a controlled manner in the tumor site. Wu et al. have designed novel azo (4,4-azobis (4-cyanovaleric acid))-functionalized magnetic functional nanoparticles to achieve light-sensitive release and combined therapy [139]. DOX was linked to IONs through the azo group (Figure 9). The system was injected to xenografted tumor mice. Then, the animals were exposed to NIR irradiation, and IONs rapidly converted the light into heat and reached the critical fracture temperature (43 °C), leading to the breakage of the azo linker and burst release of DOX. The results demonstrated that chemo-photothermal therapy using IONs–azo–DOX was more effective in tumor-suppression than chemotherapy or photothermal therapy alone.

Magnetic liposomes functionalized with hyaluronic acid were used as the vehicle for targeted delivery and triggered release of docetaxel, an anticancer drug, in human breast cancer cells [140]. Under NIR laser irradiation, the liposomes can reach 46.7 °C in 10 min, and they release over 20% more of the drug than the non-irradiated liposomes.

The cell nucleus is recognized as the ideal target for cancer treatment because it plays a central role in genetic information and the transcription machinery reside. Peng et al. conjugated transferrin and TAT peptide (TAT: YGTRKKRRQRRR) to IONs [102]. Such nanoparticles exhibited high photothermal conversion (~37%) and considerable photothermal stability.

All of the studies described above have used the first biological near-infrared window. The structure of magnetite, that is, inverse spinel, implies that Fe(II) and Fe(III) ions in the octahedral sites of the crystal can produce an intervalence charge transfer that gives rise to a second near-IR region at 1000–1350 nm. At this window, the radiation is less affected by scattering losses and is able to achieve deeper penetration. In this way, Huang et al. fabricated magnetite cluster-structured nanoparticles (ION-CNPs) that exhibited low absorbance at a range of 750–900 nm and a progressive evolution in the second NIR region [141]. The photothermal conversion efficiency of these nanoparticles was 20.8%, and using a 1064 nm laser at a power density of 380 mW·cm^−2^, ION-CNPs at 375 ppm in iron led to a rise in the water temperature from 25 °C to 58 °C. Moreover, the nanoparticles showed their ability to magnetically assist the photothermal ablation of cervical cancer cells after irradiation with a laser of the second near-IR region. In another study, a double layer of gold/silver alloy was put on the surface of truncated octahedral IONs. By controlling the distance between the layers, the NIR absorption from 650 to 1300 nm can be tuned [51].

Finally, it is interesting to remark the possibility of reactions between the nanoparticles and the adjacent polymers or biomolecules when a laser is applied. These reactions can affect the physicochemical properties of the nanoparticles. It is known that when nanoparticles are exposed to an in vivo system, a new adsorption layer of proteins, the so-called protein corona, is formed by the biomolecules present in the biological milieu [142]. On the other hand, the presence of polymers coating the particle, concretely the PEG, can alter the cellular uptake of the nanomaterial [129]. When external photonic stimuli are applied, some new kinds of interactions can appear, as has been demonstrated with magnetic nanoparticles embedded in polymer matrices after irradiation with a 780 nm multiphoton laser [143,144].

## 3. Conclusions and Perspectives

PTT is a light-triggered, minimally non-invasive, and effective modality for cancer treatment, which is based on the transduction of light into heat by NIR-light sensitive nanoparticles. Several types of nanoparticles, the so-called photothermal agents, can convert light into heat. However, the nanoparticle platforms used exhibited variations in efficacy and toxicity. As reliable biosafety in the human body is the main requisite for any treatment of cancer, IONs appear as promising photothermal agents. IONs can be used either in combination with other photothermal agents or by themselves. They present a number of advantages, such as excellent magnetic properties, good compatibility, and absence of toxicity. The magnetic properties of IONs allow them to be magnetically targeted and used as contrast agents in MRI. In the reviewed studies, IONs have proven to effectively induce site-specific cell death in both in vivo and in vitro treatment, and prevent side effects from occurring. However, IONs still suffer from certain disadvantages. The main drawback is the high dose of irradiation required for complete ablation of the tumor. In some cases, the irradiation can far exceed the safe limit for cutaneous tissues. In this review, we have found several approaches used to solve this concern. For instance, clustered IONs present an important increase in NIR absorption in relation to the non-aggregated IONs. On the other hand, the heating efficiency can be amplified by magnetic hyperthermia, giving the IONs the dual capacity to act as both magnetic and photothermal agents. Further studies with IONs must address the preparation of platforms with high thermal efficiency and total absence of side harmful effects.

## Abbreviations

ACB	accelerated blood clearance
AMF	alternating magnetic field
BSA	Bovine serum albumin
CNP	Cluster-structured nanoparticles
DMSA	meso-2,3-dimercaptosuccinic acid
DOX	doxorubicin
DSPE	distearoyl-sn-glycero-3-phosphoetanolamine
EGFR	epidermal growth factor receptor
EPR	enhanced permeability and retention
FDA	Food and Drug Administration
HSA	human serum albumin
ICG	indocyanine green
ION	iron oxide nanoparticle
LSPR	local surface plasmon resonance
MRI	magnetic resonance imaging
NIR	near-infrared
PA	photothermal agent
PDT	photodynamic therapy
PEG	poly(ethylene) glycol
PPY	polypyrrole
PTT	photothermal therapy
RBC	red blood cells
